# Aqueous Solutions of Oil-Soluble Polyglycerol Esters: Structuring and Emulsifying Abilities

**DOI:** 10.3390/molecules30234507

**Published:** 2025-11-22

**Authors:** Rumyana Stanimirova, Mihail Georgiev, Krassimir Danov, Jordan Petkov

**Affiliations:** 1 Department of Chemical & Pharmaceutical Engineering, Faculty of Chemistry & Pharmacy, Sofia University “St. Kliment Ohridski”, 1164 Sofia, Bulgaria; rs@lcpe.uni-sofia.bg (R.S.); mtg@lcpe.uni-sofia.bg (M.G.); jordan.petkov@arxada.com (J.P.); 2 Centre of Competence “Smart Mechatronics, Eco- and Energy Saving Systems and Technologies”, 1164 Sofia, Bulgaria; 3 Arxada, Hexagon Tower, Crumpsall Vale, Blackley, Greater Manchester, Manchester M9 8GQ, UK; 4 Biological Physics, School of Physics and Astronomy, The University of Manchester, Schuster Building, Oxford Road, Manchester M13 9PL, UK

**Keywords:** oil-soluble polyglycerol esters (PGE), structures in PGE aqueous solutions, emulsifying ability of PGE, rheology of PGE aqueous solutions and emulsions

## Abstract

The polyglycerol esters (PGEs) of fatty acids have a wide range of HLB values and applications in diverse industries, such as pharmaceuticals and cosmetics. While the physicochemical properties of oil-soluble PGEs dissolved in oil phases are well studied in the literature, there is no information on their structuring in aqueous phases and emulsifying abilities. We combined rheological and differential scanning calorimetry (DSC) measurements and microscopy observations to characterize the dependence of oil-soluble PGE structuring in aqueous phases on the PGE concentration, the temperature of solution homogenization, and the PGE molecular structure. Excellent correlations between the considerable changes in solution viscosity and the temperatures of the two endo- and exothermic peaks in the DSC thermograms are observed. Single-tail PGE molecules, which have a higher number of polyglycerol units, are better organized in networks, and the viscosity of their aqueous solutions is higher compared to that of the respective double-tail PGE molecules. PGEs exhibit good emulsifying ability and the viscosity of the produced emulsions at room temperature can differ by orders of magnitudes depending on the temperature of emulsification. The reported properties of oil-soluble PGEs could be of interest for increasing the range of their applicability in practice.

## 1. Introduction

Polyglycerol esters (PGEs) of fatty acids are multifunctional, nonionic surfactants obtained by esterification of polyglycerol and fatty acids [1,2,3,4,5,6,7]. Their properties depend on the degree of glycerol polymerization and the fatty acid chain length, which determine the molecular weight, solubility, and type of ester. PGEs are characterized by a wide range of hydrophile–lipophile balance (HLB) values (from 3 to 14), and they can be oil-soluble, water-soluble, or partially soluble in both aqueous and oil phases depending on their HLB. This versatility makes them widely used molecules in the food [1,2,3,8,9,10,11,12,13,14], pharmaceutical [15,16,17], and cosmetic industries [18,19,20,21], enabling emulsion stabilization, rheology modulation, and encapsulation of bioactive compounds.

Polyglycerol esters are known for their biodegradability, biocompatibility, non-toxicity, renewable sourcing, and environmental compatibility, and they have been proposed as potential green alternatives to polyethylene glycol-based nonionic surfactants. Their safety is well established: EFSA and Fiume et al. confirmed safety in foods and cosmetics [8,9,10]; Fröhlich et al. [22] reported no pulmonary effects; and Hanno et al. [23] demonstrated the possibility of sustainable production from rice bran oil.

Oil-soluble PGEs are widely used as co-emulsifiers, structuring agents, and crystallization modifiers, and they can serve as vehicles for producing nanoemulsions or nanoparticles for nutrients and drug delivery. In the food industry, oil-soluble PGEs suppress the crystallization of saturated lipids [24,25,26] and improve fat crystal structure, air retention, and stability in dairy products [27,28]. PGEs enhance the rheological behavior of oleogels at different temperatures [29]. They structure oils for topical formulations and organogels [30,31], and their fatty acid compositions strongly influence the efficiency of the formed structures—the long-chain PGEs outperform medium-chain PGEs and glycerol mono-stearate [32]. The crystallization and viscoelasticity of structured oils and gels produced with polyglycerol esters [33,34,35] have been studied by different experimental techniques (differential scanning calorimetry, microscopy, advanced rheology, etc.). The addition of oil-soluble polyglyceryl-10 mono-stearate to cetyl isooctanoate forms an interdigitated lamellar structure that stabilizes emulsions [36]. In Refs. [37,38], the authors reported that the generation of a lamellar phase together with a liquid-crystal phase (Lc) and a sponge phase (L3) in the solutions leads to the formation of nanoemulsions with droplet sizes less than 50 nm during low-energy emulsification. The rheological functionality of emulsions stabilized with PGEs can be improved by increasing interfacial elasticity [39,40]. Hashizaki et al. [41] observed a formation of reverse wormlike micelles in lecithin/PGE/oil systems, considerably affecting the viscoelasticity of solutions. The respective growth of formed reverse wormlike micelles, shear-thickening rheological behavior, and zero-shear viscosity depends on the PGE concentration, the degree of polyglycerol polymerization, and the HLB value.

In pharmaceuticals and cosmetics, PGEs serve as delivery systems: PGE micelles enhance solubility and uptake of coenzyme Q10 [42]; stable curcumin and insulin carriers have been developed with caseinate and lipid nanocarriers [43,44]; and nanoscale PGE carriers have been investigated in several studies [45,46,47]. Stable ethanol-containing O/W emulsions with high ethanol content have been studied in Ref. [48]. The main properties and potential applications of PGEs are summarized in the recent review [49].

In our previous study [50], we studied the colloidal, interfacial, and foam properties of water-soluble PGE aqueous solutions. Our goal in the present study is to characterize the irreversible bulk structuring in aqueous solutions of oil-soluble PGEs (polyglyceryl-10 di-palmitate, polyglyceryl-6 di-stearate, polyglyceryl-3 mono-stearate, and polyglyceryl-10 mono-stearate) depending on the temperature of homogenization and concentration, the number of glycerol units, the chain length, and number of chains in the molecules. The combination of the rheological measurements, differential scanning calorimetry (DSC), and microscopy observations provides detailed information on the consistency and power-law index of the rheological model for the viscosity, the temperatures of the formations of different bulk structures, and their types. The possibility of using the effect of PGE structuring in aqueous phases to produce stable oil-in-water emulsions with high or low viscosities is discovered.

## 2. Results

### 2.1. Rheology in a Steady Shear Regime of PGE Solutions

**Figure 1:**
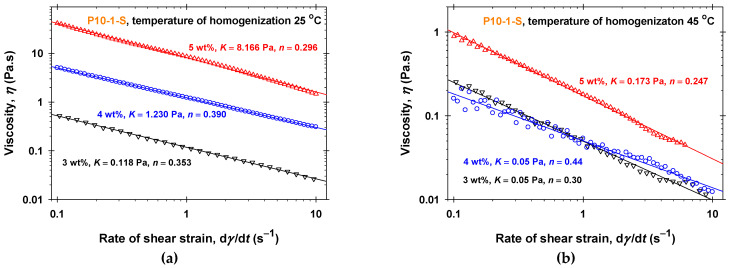
Dependence of the viscosity, *η*, on the shear rate, d*γ*/d*t*, and the concentration of P10-1-S in aqueous solutions measured at *T* = 25 °C: (**a**) 25 °C temperature of homogenization; (**b**) 45 °C temperature of homogenization. The solid lines correspond to the best-fit results using the Ostwald-de Waele rheological model with the parameters listed in Table S1.

The effect of the polyglycerol ester concentration, *C*, and the temperature of homogenization, *T*_hom_, on the dependence of the viscosity (*η* measured at 25 °C) on the rate of shear strain, d*γ*/d*t*, for P10-1-S and P10-2-P aqueous solutions is illustrated in Figure 1 and Figure S1, respectively. In all cases, the Ostwald–de Waele rheological model describes well the shear-thinning properties of the P10-1-S aqueous solutions with power-law indexes 0.24 < *n* < 0.44, see Table S1. The consistency, *K*, gradually increases with increasing P10-1-S concentrations (from 0.118 ± 0.005 Pa to 8.166 ± 0.005 Pa for *T*_hom_ = 25 °C and from 0.05 ± 0.01 Pa to 0.173 ± 0.006 Pa for *T*_hom_ = 45 °C). The rheological responses of P10-2-P aqueous solutions (Figure S1) are considerably lower compared to the respective P10-1-S solutions (Figure 1). In the case of P10-2-P, the measured viscosities for concentrations of 3 wt% and 4 wt% and *T*_hom_ = 45 °C are lower than 0.1 Pa·s. For 3 wt% and *T*_hom_ = 25 °C, the viscosity vs. shear rate data at low shear rates are close to those for 4 wt%, but for d*γ*/d*t* > 2 s^−1^, the values of the viscosities become about two times lower. The decrease in the temperature of homogenization from 45 °C (Figure 1b and Figure S1b) to 25 °C (Figure 1a and Figure S1a) leads to more than 10 times higher viscosities of both types of PGE solutions at a given concentration and to a considerable increase in the consistency, *K*. The rise in the PGE concentration, *C*, from 3 wt% to 5 wt% affects the rheological response more pronouncedly for *T*_hom_ = 25 °C than at the higher homogenization temperature. Thus, the P10-1-S and P10-2-P structures formed at 45 °C are more easily broken down under intensive shearing, while those at 25 °C tend to form networks under homogenization.

**Figure 2:**
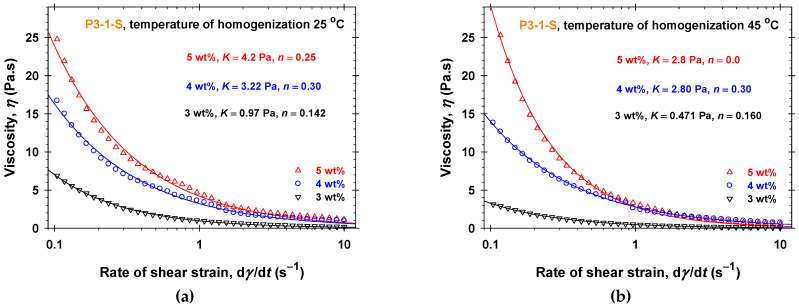
Dependence of the viscosity, *η*, on the shear rate, d*γ*/d*t*, and the concentration of P3-1-S in aqueous solutions measured at *T* = 25 °C: (**a**) 25 °C temperature of homogenization; (**b**) 45 °C temperature of homogenization. The solid lines correspond to the best-fit results using the Ostwald–de Waele rheological model with the parameters listed in Table S1.

The molecular structures of oil-soluble PGEs play an important role in the structuring in aqueous phases. P10-1-S and P10-2-P have equal numbers of glycerol units and approximately equal chain lengths, but different numbers of chains. Our results (Figure 1, Figure 2, Figures S1 and S2) show that the single-tail ester (P10-1-S) molecules are better organized in networks than the double-tail ester (P10-2-P), and the measured viscosities of P10-1-S aqueous solutions at both temperatures of homogenization are relatively high. Note that not only the chain length but also the type of hydrophobic chain in PGE molecules plays an important role in the structuring in aqueous solutions. For example, P10-1-O (polyglyceryl mono-oleate ester) solutions have low viscosities even at 25 °C temperature of homogenization [50], compared to those of P10-1-S solutions (Figure 1a).

To prove the crucial effect of the number of glycerol units in the oil-soluble PGEs on network formation, we performed analogous rheological experiments using P3-1-S aqueous solutions (Figure 2). As is evident from Figure 2, the rheological response of P3-1-S aqueous solutions practically does not depend on the temperature of homogenization. The P3-1-S solutions have pseudoplastic shear-thinning behavior and for both *T*_hom_—the highest viscosities comparable to all other studied PGEs. The consistency, *K*, increases with increasing P3-1-S concentrations (from 0.97 ± 0.01 Pa to 4.2 ± 0.1 Pa for *T*_hom_ = 25 °C and from 0.0471 ± 0.003 Pa to 2.80 ± 0.06 Pa for *T*_hom_ = 45 °C) and the power-law indexes, *n*, are less than 1, see Table S1.

Even for double-tailed PGEs, decreasing the number of glycerol units leads to rheological responses independent of homogenization temperatures of 25 °C and 45 °C (Figure S2). Note that P6-2-S aqueous solutions at both temperatures of homogenization have higher viscosities than P10-2-P solutions (Figure S1) for all PGE concentrations. In the case of P6-2-S, the power-law indexes are approximately equal to the order of 0.5 and the consistency increases with the rise in concentration from 0.106 ± 0.002 Pa to 0.471 ± 0.009 Pa (Table S1). Thus, reducing the number of glycerol units in the PGE molecules (for P3-1-S and P6-2-S) makes the viscosity of their aqueous solutions insensitive to the homogenization temperature.

### 2.2. Differential Scanning Calorimetry Measurements

**Figure 3:**
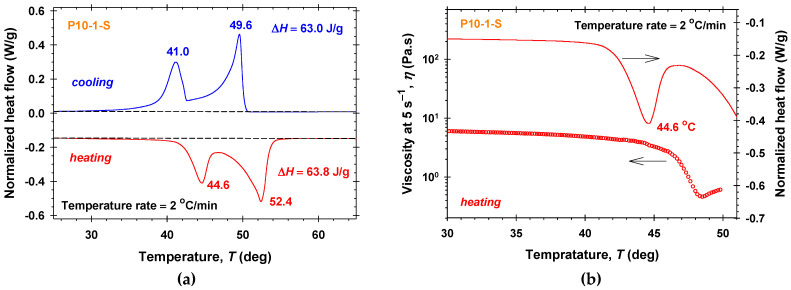
P10-1-S samples: (**a**) DSC heating and cooling thermograms; (**b**) DSC heating thermogram and the temperature dependence of the viscosity measured at 5 s^−1^.

**Figure 4:**
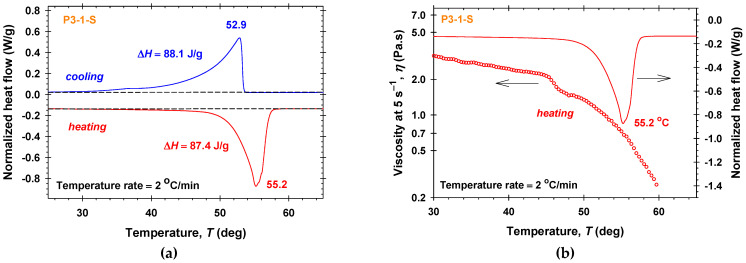
P3-1-S samples: (**a**) DSC heating and cooling thermograms; (**b**) DSC heating thermogram and the temperature dependence of the viscosity measured at 5 s^−1^.

The rheology of PGE aqueous solutions measured at 25 °C depends on the molecular structures of PGEs and on the homogenization temperature. For a deeper understanding of the physicochemical origins of these effects, we performed differential scanning calorimetry (DSC) measurements involving heating and subsequent cooling of the samples with a temperature rate of 2 °C/min. In parallel, we examined the temperature dependence of the viscosity at a constant shear rate of 5 s^−1^, following the same thermal protocol. The results obtained are summarized in Figure 3, Figure 4, Figures S3 and S5.

For P10-1-S samples (Figure 3a), two endothermic peaks appear upon heating at 44.6 °C and 52.4 °C, with a total enthalpy from the endothermic transition of Δ*H* = 63.8 J/g (Figure 3a). This indicates a two-step melting process involving solid–solid rearrangement followed by a complete melting of the stable domains. Such dual peaks are a characteristic of the fatty esters—they are typically associated with low-temperature polymorphic reorganization or crystal perfection and high-temperature melting of the stable crystalline domains. This behavior is well documented for lipids and fatty acid esters, where the polymorphism and the multistep crystallization are common [51,52]. During cooling, two exothermic peaks were observed at 49.6 °C and 41.0 °C, with a total exothermic enthalpy of Δ*H* = 63.0 J/g consistent with a two-step crystallization (primary nucleation followed by perfection). A clear correlation is observed between the DSC-detected thermal transitions and the changes in the viscosity with temperature *T* (Figure 3b). The increase in temperature above 44 °C leads to a sharp decrease in the viscosity, *η*.

Polarized light microscopy images from P10-1-S aqueous solutions heated with a temperature rate of 2 °C/min and taken from a fixed sample region are shown in Figure S4. Distinct liquid-crystalline textures characterized by changing colors are clearly observed throughout the entire sample. With increasing temperature, a noticeable change in the brightness of the crystals is detected. At *T* = 47 °C, the colors fade significantly, and the sample becomes more fluid. This change is attributed to polymorphic reorganization of the crystalline structures. The temperature of 47 °C corresponds exactly to the minimum of the absolute value of the normalized heat flow curve measured between the two endothermic peaks in Figure 3a. In the temperature range between 47 °C and 58 °C, an additional fading of the colors is detected, indicating a reorganization of the crystalline structures. Upon reaching temperature of 58.6 °C, the liquid-crystalline textures completely melt and drops appear in the sample.

In the case of P10-2-P samples (Figure S3a), the first endothermic peak in the DSC heating thermograms appears at the lower temperature of 39.3 °C and the second one at 47.9 °C. The corresponding two exothermic peaks are observed at 36.3 °C and 44.8 °C. The total enthalpies from both endothermic and exothermic processes of Δ*H* = 54.5 J/g are equal. Thus, because of the same numbers of glycerol units in P10-1-S and P10-2-P molecules, the magnitudes of the first DSC peaks for P10-1-S and P10-2-P are quite similar. The decrease in the chain length leads to lower magnitudes of the second DSC peaks, lower values of the total enthalpies, and more pronounced viscosity drops (Figure S3b).

Previous studies on polyglycerol fatty acid esters (PGFEs) [49,53] demonstrate that the glycerol degree of polymerization and the number and length of fatty acid tails strongly influence the HLB, molecular packing, and PGFE phase behavior. Smaller headgroups and/or longer saturated tails enhance the van der Waals interactions, leading to tighter packing, higher melting temperatures, and larger enthalpies. It is obvious, that in the DSC heating and cooling thermograms of P3-1-S samples (Figure 4a), the peaks at lower temperatures of about 45 °C disappear, and the absolute values of the normalized heat flow continuously increase as the temperature rise to the maximums of the absolute values of the normalized heat flow. Well-pronounced endothermic peak at 55.2 °C and exothermic peak at 52.9 °C are measured. In contrast to P10-1-S, the magnitudes of the second peaks for P3-1-S are larger compared to those for P10-1-S, and the resulting total enthalpies from both endothermic and exothermic transitions of 87.4 J/g and 88.1 J/g, respectively, are considerably higher. The decrease in the viscosity of P3-1-S solutions with temperature is observed for *T* > 44 °C (Figure 4b).

**Figure 5:**
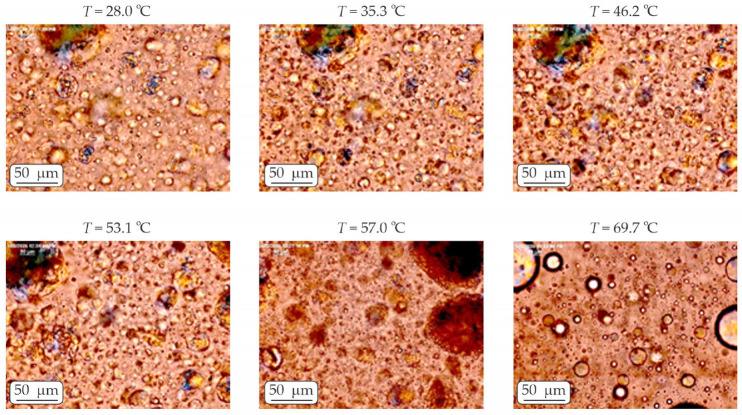
Polarized light microscopy images taken of P3-1-S aqueous solution during heating at a temperature rate of 2 °C/min. All images are captured from a fixed sample region without changing the microscopy table position. The scale bar represents 50 μm.

Polarized light microscopy images taken at different temperatures of P3-1-S aqueous solutions during heating at a temperature rate of 2 °C/min are shown in Figure 5. A larger number of liquid-crystalline structures are observed, remaining stable throughout the heating up to temperature of 53 °C. Upon subsequent heating, these ordered domains gradually melt, which is manifested by a noticeable decrease in birefringence and a fading of the vivid colors. The temperature at which this transition occurs corresponds closely to the endothermic peak observed in the DSC thermogram at 55.2 °C (Figure 4a), confirming the consistency between the optical and thermal analyses.

The DSC thermograms of P6-2-S samples (Figure S5a) are characterized by two endothermic peaks (at 43.6 °C and 51.6 °C) and two exothermic peaks (at 40.4 °C and 48.4 °C). Because of the smaller number of glycerol units in the P6-2-S molecules, the magnitudes of the normalized heat flows at the first peaks are lower than those in the case of P10-1-S. Nevertheless, the total enthalpies for double-tail molecules of P6-2-S (endothermic enthalpy of 76.1 J/g and exothermic enthalpy of 75.3 J/g) are higher than those of P10-1-S. For P6-2-S samples, the first peak in the DSC heating thermograms corresponds to the onset of the viscosity reduction (Figure S5b). The higher enthalpy indicates stronger crystalline organization, resulting in smooth changes in the rheological behavior. Thus, the decrease in the number of glycerol units in the PGE molecules (for P6-2-S) leads to the decrease in the magnitude of the first peak in the DSC thermograms corresponding to the lower temperatures (of about 40 °C), which becomes negligible for P3-1-S. Across all samples, viscosity serves as a sensitive indicator of the structural transitions taking places at different temperatures.

### 2.3. Emulsifying Ability of PGEs

It is reported in the literature [50] that the presence of polyglyceryl-10 mono-oleate ester (P10-1-O) in water and oil systems leads to a spontaneous emulsification. The corresponding oil-in-water (50:50) emulsions are stable and their behavior does not depend on the phase in which P10-1-O is initially dissolved. Below we demonstrate that oil-soluble PGEs dissolved in the water phase can be used as effective emulsifiers to produce emulsions with quite different properties (high or low viscosity, long-term stable or unstable, etc.) depending on the structuring of the PGE molecules in the water phase at different temperatures of emulsion preparation, *T*_em_. All studied emulsions below contain 15 wt% SFO, and they are stabilized only with P10-1-S, P10-2-P, P6-2-S, or P3-1-S surfactants.

**Figure 6:**
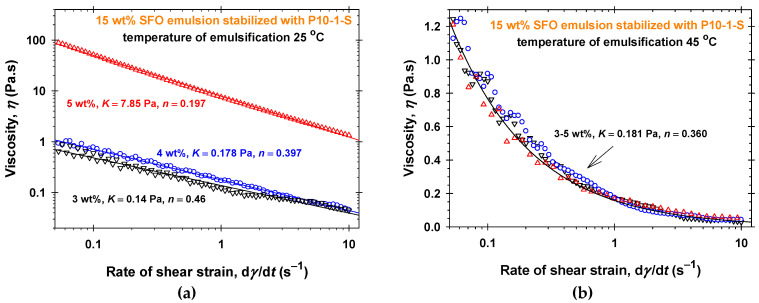
Dependence of the viscosity, *η*, of 15 wt% SFO emulsions stabilized with P10-1-S on the shear rate, d*γ*/d*t*, and the concentration of P10-1-S in the water phase measured at *T* = 25 °C: (**a**) 25 °C temperature of emulsification; (**b**) 45 °C temperature of emulsification. The solid lines correspond to the best-fit results using the Ostwald–de Waele rheological model with the parameters listed in Table S2.

**Figure 7:**
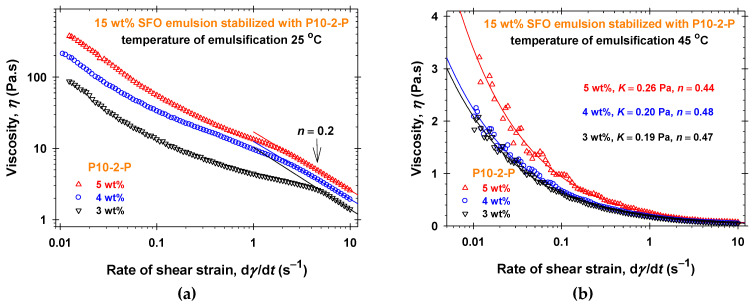
Dependence of the viscosity, *η*, of 15 wt% SFO emulsions stabilized with P10-2-P on the shear rate, d*γ*/d*t*, and the concentration of P10-2-P in the water phase measured at *T* = 25 °C: (**a**) 25 °C temperature of emulsification; (**b**) 45 °C temperature of emulsification. The solid lines correspond to the best-fit results using the Ostwald–de Waele rheological model with the parameters listed in Table S2.

The rheological behavior measured at 25 °C (viscosity vs. shear rate) of 15 wt% sunflower seed oil (SFO) emulsions stabilized with different concentrations of PGEs dissolved in the water phase is illustrated in Figure 6, Figure 7, Figures S6 and S7. For all studied PGEs and their concentrations, the emulsions were prepared at two temperatures of emulsification (*T*_em_ = 25 °C and *T*_em_ = 45 °C). The comparisons between Figure 2 and Figure S6 for P3-1-S and Figures S2 and S7 for P6-2-S show that the corresponding aqueous solutions and 15 wt% SFO emulsions at fixed PGE concentrations have the same rheological response in the frame of reproducibility errors both at different temperatures of homogenization, *T*_hom_, and emulsification, *T*_em_. The dependencies of the shear strain on the shear rate are described by the Ostwald–de Waele rheological model (Tables S1 and S2). Thus, the presence of emulsion drops (15 wt% SFO) does not affect the formed structures in the case of P3-1-S and P6-2-S. After three weeks of storage at room temperature, only emulsions stabilized with 5 wt% P3-1-S at both temperatures of emulsification are stable (Table S3). Note that the 5 wt% P3-1-S aqueous phase and emulsions have the highest viscosity at low shear rates, with *η* about 25 Pa·s at a shear rate of 0.1 s^−1^ (Figure 2, Figures S2, S6 and S7).

The increase in the number of glycerol units in the PGE molecules (P10-1-S and P10-2-P) leads to a pronounced effect of the oil drops on the rheology of emulsions. In the case of P10-1-S (Figure 1 and Figure 6), the emulsions stabilized with 5 wt% P10-1-S and prepared at *T*_em_ = 25 °C are pronouncedly more viscous than the 5 wt% P10-1-S aqueous solution homogenized at the same temperature. The increase in the temperature of emulsification, *T*_em_ = 45 °C, leads to identical rheological responses for all studied P10-1-S concentrations (Figure 6b): the emulsion viscosity is slightly higher than that of the 5 wt% P10-1-S aqueous solution homogenized at the same temperature (Figure 1b). Note that only the viscosity of emulsion stabilized with 5 wt% P10-1-S at *T*_em_ = 25 °C measured at a shear rate of 0.1 s^−1^, of about 50 Pa·s is higher than 25 Pa·s, and this emulsion is stable upon long-term storage at room temperature (Table S3). Thus, the increase in the number of polyglycerol units in the PGE molecule favors structuring in the water phase of oil-in-water emulsions.

Surprisingly, the presence of oil drops has a significant effect on the PGE structuring in the water phase for 15 wt% SFO emulsions stabilized with P10-2-P (Figure 7). For both temperatures of emulsification and all P10-2-P concentrations, the emulsions have more than 10-times higher viscosities than the respective P10-2-P aqueous solutions prepared under the same temperature conditions (Figure 7 and Figure S1). The high viscosities without plateau values (zero-shear viscosities) at low shear rates of 15 wt% SFO emulsions prepared at 25 °C and stabilized with all studied concentrations of P10-2-P (Figure 7a) are attributed to high elasticity of the complex fluids [54]. All these emulsions are stable for at least three weeks of storage (Table S3). The increase in emulsion viscosity in the case of *T*_em_ = 45 °C (Figure 7b), compared to the viscosity of the respective P10-2-P aqueous solutions (Figure S1b), is not enough to stabilize these emulsions for long periods (Table S3).

**Figure 8:**
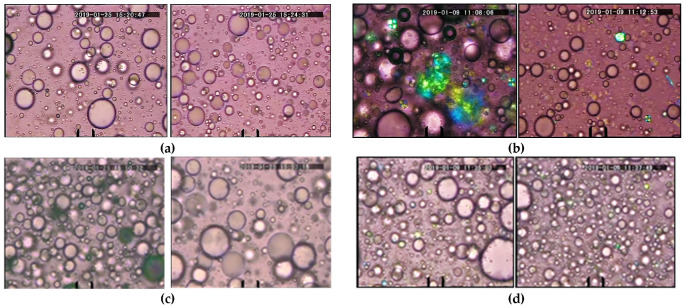
Micrographs of PGE emulsions containing 15 wt% SFO and stabilized with 5 wt% PGE dissolved in the water phase: (**a**) P10-1-S; (**b**) P3-1-S; (**c**) P10-2-P; (**d**) P6-2-S. Left images correspond to *T*_em_ = 25 °C and the right—to *T*_em_ = 45 °C. The scale bar represents 20 μm.

Micrographs of 15 wt% SFO emulsions stabilized with 5 wt% PGE dissolved in the water phase, produced at emulsification temperatures of 25 °C and 45 °C, and stored for 24 h at 25 °C, are shown in Figure 8. In the cases of P10-1-S, P3-1-S, and P10-2-P at *T*_em_ = 25 °C and P3-1-S at *T*_em_ = 45 °C, the structuring of the PGEs molecules in the water phase and at the interfaces of the oil drops is clearly visible. Note that these emulsions remained stable for at least three weeks at room temperature.

## 3. Discussion

The irreversible structuring in water of the oil-soluble PGEs depends considerably on the temperatures of homogenization and their molecular structures (Section 2). The structures formed at a given *T*_hom_ remain unchanged when cooled down to 25 °C, and the respective rheological behavior of the aqueous solutions at 25 °C becomes dependent on *T*_hom_. This peculiar property of oil-soluble PGEs allows their application to produce stable or unstable oil-in-water emulsions with controllable rheological characteristics depending on the temperature of emulsification, *T*_em_. Typical wormlike micellar solutions obey shear-thinning rheological behavior without pronounced thixotropic effects [54]. All reported results in Section 2 show that PGE aqueous solutions and PGE-stabilized emulsions also follow the shear-thinning rheology, but, as demonstrated below, with pronounced thixotropy. Thixotropy is an important factor which should be taken into account in industrial conveying systems and in tube filling processes.

**Figure 9:**
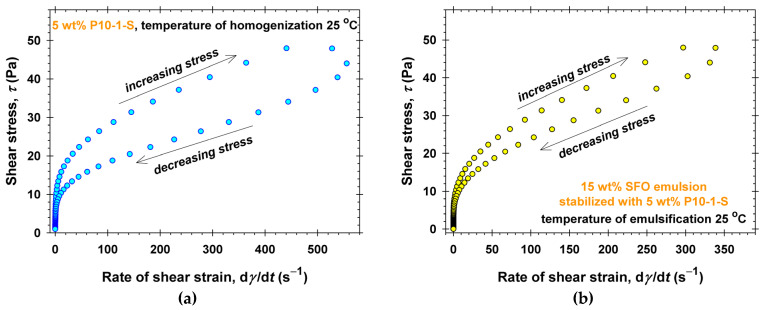
Thixotropic rheological experiments performed at 25 °C: (**a**) 5 wt% P10-1-S aqueous solutions, *T*_hom_ = 25 °C; (**b**) 15 wt% SFO emulsion stabilized with 5 wt% P10-1-S dissolved in the water phase, *T*_em_ = 25 °C.

The results from the thixotropic experiments performed with 5 wt% P10-1-S aqueous solutions and the respective oil-in-water emulsions are summarized in Figure 9. In all experiments, *T*_hom_ = *T*_em_ = 25 °C, and the emulsions are stable. First, the thixotropic loop measured for 5 wt% P10-1-S aqueous solution (Figure 9a) is noticeably more pronounced than that observed for the emulsion stabilized with the same amount of PGE (Figure 9b). Second, a shear stress of 50 Pa is reached at a shear rate of about 500 s^−1^ for the P10-1-S aqueous solution compared to a shear rate of about 350 s^−1^ for the emulsion. The respective apparent viscosities of aqueous solutions are lower than those of the emulsions, which is also confirmed by the measurements shown in Figure 1 and Figure 6. Thus, the presence of oil drops (15 wt% SFO) in the emulsions partially suppresses the thixotropy of the complex fluids.

**Figure 10:**
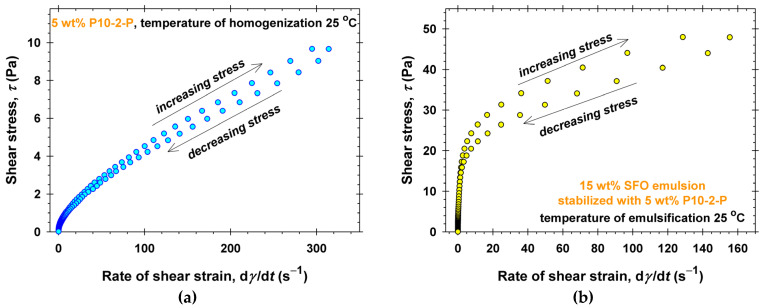
Thixotropic rheological experiments performed at 25 °C: (**a**) 5 wt% P10-2-P aqueous solutions, *T*_hom_ = 25 °C; (**b**) 15 wt% SFO emulsion stabilized with 5 wt% P10-1-S dissolved in the water phase, *T*_em_ = 25 °C.

The P10-2-P aqueous solutions have relatively lower viscosities (Figure S1), so the thixotropic experiments were performed with applied shear stresses up to 10 Pa (Figure 10a). As expected, the low apparent viscosities are accompanied by relatively weak thixotropic loops. In contrast, the viscosities of the stable 5 wt% P10-2-P stabilized emulsions at *T*_em_ = 25 °C are orders of magnitudes higher (Figure 7a), and a shear rate of about 150 s^−1^ is measured at a shear stress of 50 Pa (Figure 10b). Note that, despite the higher viscosities compared to P10-1-S emulsions, the thixotropic loop shown in Figure 10b is less pronounced. Thus, the structures formed by P10-2-P in the presence of oil drops are more stable upon shearing than those of P10-1-S.

The viscosities of 5 wt% P3-1-S aqueous solutions and emulsions are quite similar at both temperatures of homogenization and emulsifications (Figure 2 and Figure S6). Nevertheless, the thixotropic experiments (Figure S8) show that the oil drops in emulsions (15 wt% SFO) stabilize the formed structures. An applied shear stress of 50 Pa produces a shear rate of about 1000 s^−1^ for 5 wt% P3-1-S aqueous solutions at *T*_hom_ = 45 °C, while that of the emulsions at *T*_hom_ = 45 °C is lower—400 s^−1^. Thus, the corresponding apparent viscosities of emulsions at a shear stress of 50 Pa becomes about 2.5 times higher than those of the aqueous solutions. One possible explanation is that, due to the adsorption of the oil-soluble PGEs at the emulsion drop interfaces, the formed adsorption layers have different interfacial rheology (interfacial elasticity and viscosity). Moreover, our preliminary experiments show that the formed adsorption layers behave as membranes with anisotropic surface stress distribution [55,56]. Future analysis of the oil-soluble PGE adsorption layers could give a deeper understanding of the different rheological behavior of emulsions depending on the molecular structures of PGEs. 

## 4. Materials and Methods

### 4.1. Materials

We studied a wide range of oil-soluble polyglycerol esters of fatty acids with different degrees of polymerization, chain lengths, and numbers of hydrocarbon tails, provided by Arxada (Manchester, UK). PGEs are produced by esterification of a polydisperse polyglycerol mixture with naturally derived fatty acids. The polymerization of polyglycerol proceeds via a step-growth mechanism, which leads to a broad spectrum of oligomers (di-, tri-, and higher polyglycerols), see Figure 1a in Ref. [50]. As a result, the headgroup sizes in the final polyglycerol esters follow a distribution around the mean value rather than being monodisperse. The specific polyglycerol esters were as follows: Geomulse^TM^ P11 RSPO MB Pastille (termed below as P10-2-P) is a polyglyceryl-10 di-palmitate ester; Geomulse^TM^ S10 RSPO MB Pastille (P10-1-S) is a polyglyceryl-10 mono-stearate ester; Geomulse^TM^ S8 RSPO MB Pastille (P6-2-S) is a polyglyceryl-6 di-stearate ester; and Syneth^TM^ S7 K RSPO MB Pastille (P3-1-S) is a polyglyceryl-3 mono-stearate ester. With respect to the hydrophobic part: P6-2-S can contain mono-, di-, and three-stearate; P10-1-S and P3-1-S—mono-stearate and di-stearate; and P10-2-P—mono -, di-, and three-palmitate. The manufacturer confirms that the major component corresponds to the indicated ester (P3-1-S, P6-2-S, P10-1-S, P10-2-P), while small amounts of related glycerol esters can be present as by-products. All samples have been used as received, without additional purification.

The oil phase for all emulsions was sunflower seed oil (SFO) obtained from a local supplier. To remove the available impurities, SFO was purified by passing it through a column filled with Silica Gel and Florisil adsorbent. The level of purification was evaluated by measuring the interfacial tension against pure water. The oil phase was considered sufficiently purified when the interfacial tension was approximately 31 mN/m and did not decrease by more than 0.2 mN/m within 60 min. The measured viscosity of sunflower seed oil was 50 mPa·s.

All aqueous solutions were prepared using deionized water of specific resistivity 18.2 MΩ·cm, purified by Elix 3 water purification system (Millipore, Burlington, MA, USA).

### 4.2. PGE Aqueous Solutions and Protocol for Respective Emulsions’ Preparation

All aqueous solutions were prepared by weighing the required amount of PGE to achieve the desired concentration (3 wt%, 4 wt%, or 5 wt%) into a beaker and subsequently heating and stirring them in a water bath at 70 °C with a magnetic stirrer until complete dissolution. The solutions were then cooled to the target temperature and homogenized using a high-shear laboratory mixer (Silverson L4RT) at 300 rpm for 3 min. Homogenization was performed at a temperature of *T*_hom_ = 25 °C or at a higher temperature of *T*_hom_ = 45 °C. The obtained solutions were cooled to 25 °C and left overnight for equilibration.

For the emulsion preparation, purified sunflower oil (SFO) was added to the fully dissolved PGE aqueous solution to achieve a 15 wt% oil content. The mixture was stirred for 5 min at 70 °C to form a coarse emulsion, which was then cooled to the desired emulsification temperature (*T*_em_ = 25 °C or *T*_em_ = 45 °C). The emulsification was performed using the high-shear laboratory mixer at 300 rpm for 3 min at the fixed *T*_em_. The obtained emulsions were cooled to 25 °C and stored at 25 °C for 24 h before the corresponding rheological study. The long-term stability of emulsions was observed after at least three weeks storage at room temperature.

### 4.3. Methods

#### 4.3.1. Rheological Measurements

The rheological behavior of the PGE aqueous solutions and the PGE-stabilized emulsions was investigated using a rotational rheometer (Bohlin Gemini, Malvern Instruments, Malvern UK) equipped with a cone-and-plate geometry. The plate temperature was maintained at 25 °C by a Peltier control unit. A solvent trap was employed to minimize sample evaporation during measurements. For samples exhibiting paste-like consistency, a 60 mm cone with an angle of 2° and a gap distance of 70 μm was used, whereas for more fluid samples, a 40 mm cone with an angle of 4° and a gap distance of 150 μm was applied. Three types of advanced rheological tests were performed: steady shear measurements; thixotropic loop experiments; and measurements of the temperature dependence of the apparent viscosity at a fixed rate of shear strain of 5 s^−1^.

In the *steady shear regime*, we measured the apparent viscosity, *η*_app_ (the ratio between the steady shear stress, *τ*, and the rate of shear strain, d*γ*/d*t*). The steady shear rate sweeps were performed in the shear rate range from 0.01 to 10 s^−1^ using a logarithmic profile with approximately 25 data points per decade. The rheometer automatically adjusted the acquisition time at each point to ensure steady-state conditions, based on a torque stability criterion of 1% deviation. The experimental data for the apparent viscosity were fitted using Ostwald–de Waele model, see Equation (1). In this model, the shear stress, *τ*, is a power-law function of the rate of shear strain, d*γ*/d*t* [57]:

*τ* = *K* [(d*γ*/d*t*)/(d*γ*/d*t*)_ch_]*^n^*, (1)

where *K* (Pa) is the consistency, *n* is the power-law index, and (d*γ*/d*t*)_ch_ = 1 s^−1^ is the characteristic shear rate, which is not an adjustable parameter. The flow behavior index, *n*, characterizes shear-thinning or shear-thickening of the studied complex fluids: *n* = 1 corresponds to Newtonian fluids with a constant viscosity; *n* > 1 indicates shear-thickening rheological behavior; *n* < 1—shear-thinning behavior.

For *thixotropic loop experiments* [58], the applied shear stress, *τ*, was increased from 0.01 to 50 Pa and subsequently decreased to 0.01 Pa at a constant rate in a logarithmic scale. The resulting shear strains and their rates were recorded over time. Prior to these measurements, a pre-shear treatment for 20 s at d*γ*/d*t* = 10 s^−1^ was applied to minimize possible effects of sample prehistory.

The *temperature dependence of the apparent viscosity* was measured at a fixed rate of shear stress of 5 s^−1^. The 10 wt% PGE aqueous solutions were heated from 20 °C to 70 °C at a fixed temperature rate of 2 °C/min, and the corresponding values of *η*_app_ were measured.

Note that all studied systems have a non-Newtonian rheological behavior. In all experiments, we measured the apparent viscosity, *η*_app_ (the ratio between the steady shear stress, *τ*, and the rate of shear strain, d*γ*/d*t*). For simplicity, we use the term viscosity and the notation *η* instead of apparent viscosity and *η*_app_, respectively.

#### 4.3.2. Differential Scanning Calorimetry (DSC)

The differential scanning calorimetry (DSC) measurements were performed with a Discovery DSC 250 system (TA Instruments, New Castle, DE, USA). For each run, approximately 10–20 mg of the PGE sample was weighed into an aluminum hermetic pan and sealed with an aluminum lid using the TZero sample press. The sealed pan was placed into the DSC oven, where the thermal protocol was carried out. The procedure included heating the samples from 20 °C to 70 °C at a constant temperature rate of 2 °C/min, followed immediately by cooling from 70 °C to 20 °C at the same temperature rate. The resulting thermograms were analyzed using the integrated functions of the Trios software package (TA Instruments, New Castle, DE, USA) to obtain the total enthalpies from the endothermic and exothermic transitions, Δ*H*. Microscopic images of 10 wt% PGE aqueous solutions in polarized light taken before each DSC experiments are shown in Figure S9.

#### 4.3.3. Optical Observations

The structuring of the PGE solutions and the respective emulsions was observed by an Axioplan microscope (Zeiss, Oberkochen, Germany) equipped with Epiplan objectives (20×, and 50×) and connected to a CCD camera with digital recording capabilities. A small portion of each sample was taken with a pipette, placed on a microscope slide, and covered with a cover slip. The observations were made using both transmitted and polarized light. The microscopy images were taken at a temperature of 25 °C.

For visualization of structural changes in the studied samples at different temperatures, we used an optical microscope (AxioImiger, Zeiss, Oberkochen, Germany) equipped with a temperature-controlled cell, allowing precise regulation of the sample temperature. The sample was heated from 26 °C to 70 °C at a constant rate of 2 °C/min. Polarized light microscopy was used to monitor structural changes during heating. All images were recorded at the same sample position to ensure consistency of observation.

## 5. Conclusions

The oil-soluble polyglycerol esters (polyglyceryl-10 mono-stearate ester, P10-1-S; polyglyceryl-3 mono-stearate ester, P3-1-S; polyglyceryl-6 di-stearate ester, P6-2-S; polyglyceryl-10 di-palmitate ester, P10-2-P) dissolved in water and cooled down to 25 °C manifest an irreversible structuring depending on their molecular structures and the temperatures of homogenization, *T*_hom_. The differential scanning calorimetry (DSC) analysis and the viscosity measurements of PGE aqueous solutions reveal two thermal transitions for P10-1-S and P10-2-P, accompanied by a sharp viscosity drop upon heating. Reducing the number of glycerol units in the PGE molecules (for P6-2-S) diminishes the magnitude of the first low-temperature DSC peak (of about 40 °C), which disappears for P3-1-S.

The structures formed in the PGE aqueous solutions during homogenization at temperatures below or above the first DSC transition remain unchanged after cooling down to 25 °C. This property of PGE aqueous solutions results in a considerable increase in the solution viscosity for P10-1-S and P10-2-P at *T*_hom_ = 25 °C compared to those measured for solutions homogenized at 45 °C. In contrast, the viscosities of P6-2-S and P3-1-S aqueous solutions are independent of *T*_hom_. In all cases, the rheological behavior (stress vs. strain) corresponds to the shear-thinning Ostwald–de Waele law. Excellent agreement between the DSC, optical, and rheological observations is achieved.

The irreversible structuring ability of the oil-soluble PGEs dissolved in water makes them good emulsifiers for the production of oil-in-water emulsions with tunable rheology and stability. The rheological properties of 15 wt% sunflower seed oil (SFO) emulsions stabilized with PGEs, prepared at different temperatures, *T*_em_, and cooled to 25 °C correlate well with the rheology of the corresponding PGE aqueous solutions. The presence of 15 wt% SFO drops in the emulsions does not significantly change the PGE structuring in the water phase but partially suppresses the thixotropy. All emulsions having viscosity at low shear rates above 25 Pa·s are stable. The only exception is the emulsions stabilized with P10-2-P at *T*_em_ = 25 °C. Even at the lowest studied concentration of 3 wt% P10-2-P, the emulsion viscosity at 0.01 s^−1^ is higher than 100 Pa·s, but the viscosity of the corresponding P10-2-P aqueous solution is 0.2 Pa·s. This observation suggests that not only the structuring but also the interfacial rheology of PGE adsorption layers on the drops could play a role in rheology and stability. The formed PGE adsorption layers seem to behave as membranes with anisotropic surface stress distribution, considerable interfacial elasticity and viscosity. Future analysis of the oil-soluble PGE adsorption layers could give a deeper understanding of the properties of emulsions depending on the PGE molecular structures.

The reported properties of the oil-soluble PGEs dissolved in water could be of interest for increasing the range of their applicability in practice.

## Data Availability

Data are contained within the article or the Supplementary Material. The original contributions presented in this study are included in the article/Supplementary Material. Further inquiries can be directed to the corresponding authors.

## Supplementary Materials

The following supporting information can be downloaded at: https://www.mdpi.com/article/10.3390/molecules30234507/s1. Figure S1: Dependence of the viscosity on the shear rate and the concentration of P10-2-P in aqueous solutions measured at *T* = 25 °C: (a) 25 °C temperature of homogenization; (b) 45 °C temperature of homogenization. Figure S2: Dependence of the viscosity on the shear rate and the concentration of P6-2-S in aqueous solutions measured at *T* = 25 °C: (a) 25 °C temperature of homogenization; (b) 45 °C temperature of homogenization. Figure S3: P10-2-P samples: (a) DSC heating and cooling thermograms; (b) DSC heating thermogram and the temperature dependence of the viscosity measured at 5 s^−1^. Figure S4: Polarized light microscopy images of P10-1-S aqueous solution during heating at a temperature rate of 2 °C/min. Figure S5: P6-2-S samples: (a) DSC heating and cooling thermograms; (b) DSC heating thermogram and the temperature dependence of the viscosity measured at 5 s^−1^. Figure S6: Dependence of the viscosity of 15 wt% SFO emulsions stabilized with P3-1-S on the shear rate and the concentration of P3-1-S in the water phase measured at *T* = 25 °C: (a) 25 °C temperature of emulsification; (b) 45 °C temperature of emulsification. Figure S7: Dependence of the viscosity of 15 wt% SFO emulsions stabilized with P6-2-S on the shear rate and the concentration of P6-2-S in the water phase measured at *T* = 25 °C: (a) 25 °C temperature of emulsification; (b) 45 °C temperature of emulsification. Figure S8. Thixotropic rheological experiments performed at 25 °C: (a) 5 wt% P3-1-S aqueous solutions, *T*_hom_ = 45 °C; (b) 15 wt% SFO emulsion stabilized with 5 wt% P3-1-S dissolved in the water phase, *T*_em_ = 45 °C. Figure S9: Microscopic images of 10 wt% PGE aqueous solutions in polarized light: (a) P3-1-S; (b) P6-2-S; (c) P10-1-S; (d) P10-2-P. Table S1: Best-fit rheological parameters of the Ostwald–de Waele model for the studied PGE aqueous solutions prepared at different temperatures of homogenization and concentrations. Table S2: Best-fit rheological parameters of the Ostwald–de Waele model for the studied PGE emulsions prepared at different temperatures of emulsification and concentrations. Table S3: Stability of emulsions containing 15 wt% SFO and different concentrations of PGE in the water phase, evaluated three weeks after preparation at both temperatures of emulsification.
