# Supplementary material for: Aqueous Solutions of Oil-Soluble Polyglycerol Esters: Structuring and Emulsifying Abilities
Source: Molecules. 2025 Nov 22;30(23):4507. doi: 10.3390/molecules30234507 (PMC12693093; doi:10.3390/molecules30234507)
Supplement: Supplementary file 1 [file molecules-30-04507-s001.zip › molecules-3989376-supplementary.pdf]

Supplementary materials

for the article

Aqueous Solutions of Oil-Soluble Polyglycerol Esters: Structuring and Emulsifying Abilities

Rumyana Stanimirova [1,2], Mihail Georgiev [1,2], Krassimir Danov [1,2,*] and Jordan Petkov [1,2,3,4]

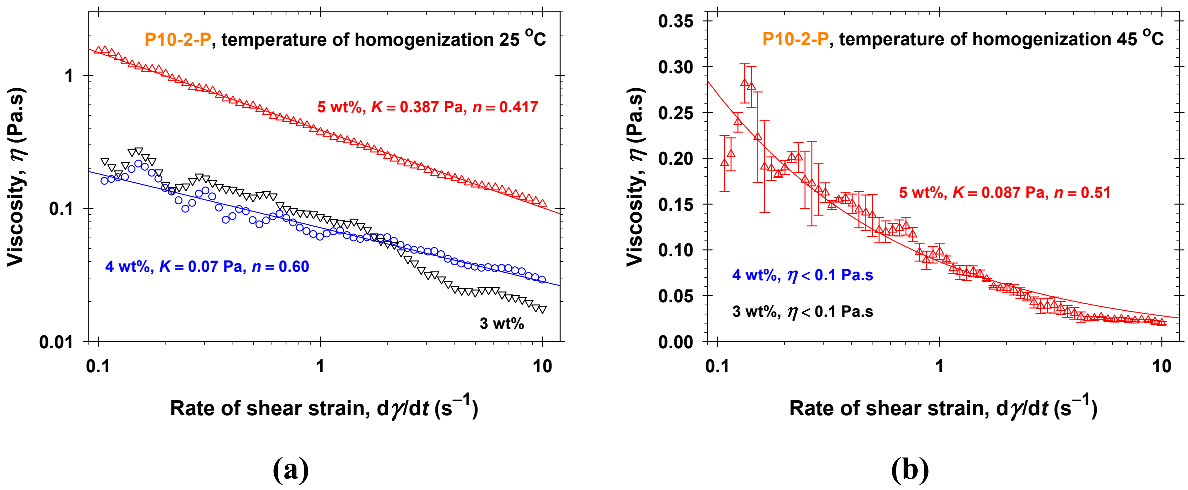

Figure S1. Dependence of the viscosity, η, on the shear rate, $d\gamma/dt$, and concentration of P10-2-P in aqueous solutions measured at T = 25 °C: (**a**) 25 °C temperature of homogenization; (**b**) 45 °C temperature of homogenization. The solid lines correspond to the best-fit results using the Ostwald–de Waele rheological model with the parameters listed in Table S1.

Figures S1 and S2 show the dependencies of the viscosity on the shear rate for P10-2-P and P6-2-S aqueous solutions (measured at temperature of 25 °C) on the PGE concentration, C, and the temperature of homogenization, T_{hom}. The solid lines therein correspond to the best-fit results using the Ostwald–de Waele model with parameters and the respective regression coefficients (r.c.) listed in Table S1. In the case of P10-2-P, the measured viscosities for concentrations of 3 wt% and 4 wt% and T_{hom} = 45 °C are lower than 0.1 Pa·s. For 3 wt% and T_{hom} = 25 °C, the viscosity vs. shear rate data at low shear rates are close to those for 4 wt%, but for $d\gamma/dt > 2$ s^{-1}, the values of the viscosities become about two times lower. This set of experimental data (P10-2-P, C = 3 wt%, T_{hom} = 25 °C) is not processed with the Ostwald–de Waele rheological model. In contrast, all studied P6-2-S aqueous solutions (Figure S2) are more viscous compared to P10-2-P, and the Ostwald–de Waele model describes the respective rheological data well (Figure S2 and Table S1).

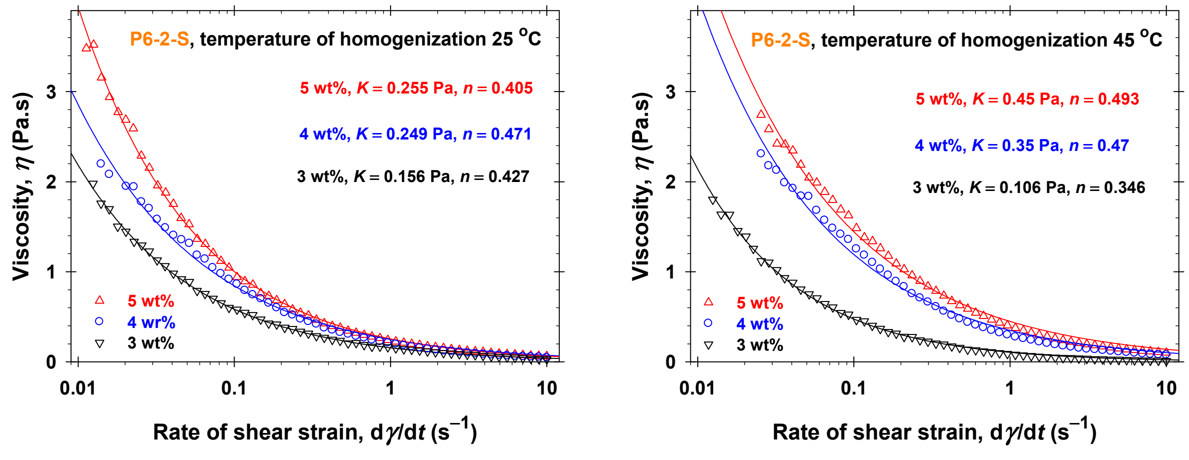

(a) **(b)**

Figure S2. Dependence of the viscosity, η, on the shear rate, $d\gamma/dt$, and concentration of P6-2-S in aqueous solutions measured at T = 25 °C: (**a**) 25 °C temperature of homogenization; (**b**) 45 °C temperature of homogenization. The solid lines correspond to the best-fit results using the Ostwald–de Waele rheological model with the parameters listed in Table S1.

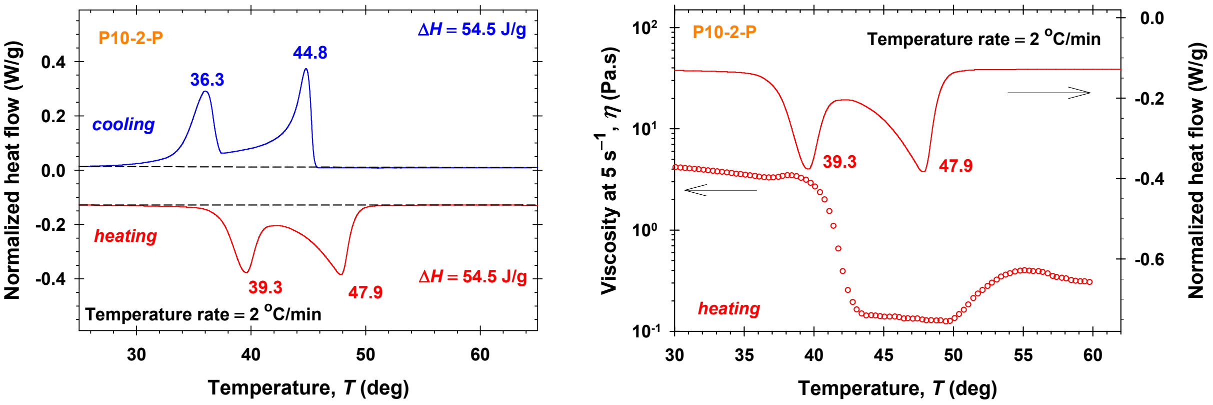

(a) **(b)**

Figure S3. P10-2-P aqueous solution: (**a**) DSC heating and cooling thermograms; (**b**) DSC heating thermogram and the temperature dependence of the viscosity measured at 5 s^{-1}.

Figures S3 and S5 summarizes the experimental results for the DSC heating and cooling thermograms of P10-2-P and P6-2-S aqueous solutions and the respective values of the viscosity measured at 5 s^{-1}. Two well-pronounced peaks of the normalized heat flows for both endothermic and exothermic processes are detected for P10-2-P (Figure S3a). The sharp viscosity decrease occurs at the temperature of 39.3 °C corresponding to the position of the first peak in the DSC heating thermogram (Figure S3a).

In the case of P6-2-S aqueous solutions, two peaks of the normalized heat flow are again visible (Figure S5a). Note that the

magnitudes of the first peaks corresponding to lower temperatures are less pronounced, while those at higher temperatures have greater absolute values than those of P10-2-P. The viscosity decreases considerably with the rise in temperature above 42 °C. For more details, see the main text.

Table S1. Best-fit rheological parameters of the Ostwald–de Waele model for the studied PGE aqueous solutions prepared at different temperatures of homogenization, T_{hom}, and concentrations, C. The temperature of all rheological measurements is 25 °C.

C **(wt%)**	***n*** **(–)**	***K*** **(Pa)**	**r.c.**	***n*** **(–)**	***K*** **(Pa)**	**r.c.**
	T_{hom} = 25 °C			T_{hom} = 45 °C		
			P10-1-S			
3 wt%	0.353 ± 0.003	0.118 ± 0.005	0.9996	0.30 ± 0.01	0.05 ± 0.01	0.9971
4 wt%	0.390 ± 0.001	1.230 ± 0.001	0.9999	0.44 ± 0.01	0.05 ± 0.01	0.9889
5 wt%	0.296 ± 0.001	8.166 ± 0.005	0.9991	0.247 ± 0.005	0.173 ± 0.006	0.9986
			P3-1-S			
3 wt%	0.142 ± 0.007	0.97 ± 0.01	0.9994	0.160 ± 0.004	0.471 ± 0.003	0.9998
4 wt%	0.30 ± 0.01	3.22 ± 0.06	0.9975	0.30 ± 0.01	2.80 ± 0.06	0.9972
5 wt%	0.25 ± 0.01	4.2 ± 0.1	0.9966	0.00 ± 0.03	2.8 ± 0.1	0.9942
			P10-2-P			
3 wt%	–	–	–	–	–	–
4 wt%	0.60 ± 0.01	0.07 ± 0.01	0.9791	–	–	–
5 wt%	0.417 ± 0.003	0.387 ± 0.005	0.9989	0.51 ± 0.02	0.087 ± 0.003	0.9712
			P6-2-S			
3 wt%	0.427 ± 0.004	0.156 ± 0.002	0.9995	0.346 ± 0.006	0.106 ± 0.002	0.9989
4 wt%	0.471 ± 0.009	0.249 ± 0.008	0.9962	0.47 ± 0.01	0.35 ± 0.01	0.9956
5 wt%	0.405 ± 0.005	0.255 ± 0.005	0.9991	0.493 ± 0.008	0.45 ± 0.01	0.9966

To visualize the structural changes in PGE aqueous solutions at different temperatures, we carried out experiments using an optical microscope equipped with a temperature-controlled cell that allows temperature variation at different rates. The samples were heated at a temperature rate of 2 °C/min within the temperature range from 26 °C to 70 °C and the images were taken under polarized light at a fixed sample region without changing the microscopy table position. Figure S4 summarizes the polarized light microscopy images of the P10-1-S aqueous solution during heating taken at different temperatures.

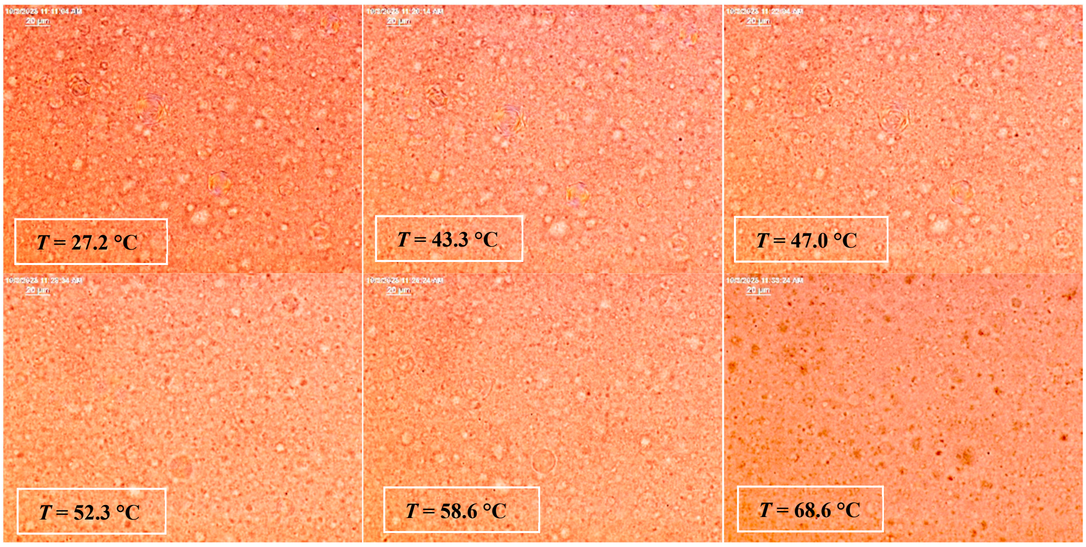

Figure S4. Polarized light microscopy images of P10-1-S aqueous solution during heating at a temperature rate of 2 °C/min. All images are captured from a fixed sample region without changing the microscopy table position.

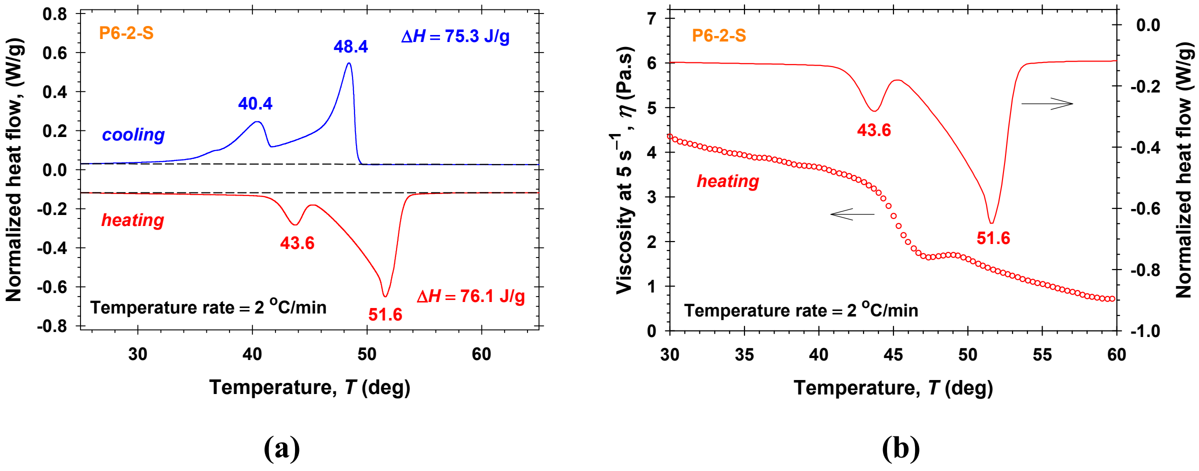

Figure S5. P6-2-S aqueous solution: (**a**) DSC heating and cooling thermograms; (**b**) DSC heating thermogram and the temperature dependence of the viscosity measured at 5 s^{-1}.

Figures 6, 7, S6, and S7 summarize the results from the rheological measurements of the viscosity vs. shear rate in the case of 15 wt% SFO emulsions stabilized with different concentrations, *C*, of the used PGEs dissolved in the aqueous phase. The temperature of the rheological experiments was fixed at 25 °C. Two temperatures of emulsification, T_{em}= 25 °C and T_{em}= 45 °C, were used in order to compare the effect of the PGE structures on the stability of emulsions. For most of the systems, the dependence of the shear stress on the rate of the shear strain is well-described using the Ostwald–de Waele model except for the P10-2-P stabilized emulsions produced at T_{em} = 25 °C. The values of the best-fit parameters and the respective regression coefficients (r.c.) are listed in Table S2.

Table S2. Best-fit rheological parameters of the Ostwald–de Waele model for the studied PGE emulsions prepared at different temperatures of emulsification, T_{em}, and concentrations, C. The temperature of all rheological measurements is 25 °C.

C **(wt%)**	***n*** **(–)**	***K*** **(Pa)**	**r.c.**	***n*** **(–)**	***K*** **(Pa)**	**r.c.**
		T_{em} = 25 °C			T_{em} = 45 °C	
			P10-1-S			
3 wt%	0.46 ± 0.01	0.14 ± 0.02	0.9878			
4 wt%	0.397 ± 0.005	0.178 ± 0.007	0.9980	0.360 ± 0.008	0.181 ± 0.003	0.9909
5 wt%	0.197 ± 0.003	7.85 ± 0.01	0.9995			
			±			
3 wt%	0.366 ± 0.004	1.89 ± 0.01	0.9993	0.311 ± 0.005	1.73 ± 0.02	0.9989
4 wt%	0.33 ± 0.01	2.10 ± 0.04	0.9953	0.32 ± 0.01	2.64 ± 0.05	0.9963
5 wt%	0.36 ± 0.01	4.0 ± 0.1	0.9909	0.28 ± 0.02	4.4 ± 0.1	0.9873
			P10-2-P			
3 wt%	–	–	–	0.47 ± 0.02	0.19 ± 0.02	0.9609
4 wt%	–	–	–	0.48 ± 0.02	0.20 ± 0.01	0.9746
5 wt%	–	–	–	0.44 ± 0.01	0.26 ± 0.01	0.9947
			P6-2-S			
3 wt%	0.22 ± 0.01	0.244 ± 0.003	0.9983	0.23 ± 0.01	0.314 ± 0.003	0.9991
4 wt%	0.20 ± 0.01	0.237 ± 0.003	0.9987	0.109 ± 0.005	0.304 ± 0.003	0.9990
5 wt%	0.17 ± 0.01	0.280 ± 0.003	0.9990	0.23 ± 0.01	0.313 ± 0.003	0.9996

The comparison between Figures 2 and S6 shows that P3-1-S aqueous solutions and corresponding 15 wt% SFO emulsions at fixed P3-1-S concentrations have the same rheological response in the frame of reproducibility errors both at different temperatures of homogenization, T_{hom}, and emulsification, T_{em}. As should be, the viscosity increases with the increase in the P3-1-S concentration, C. Thus, the presence of emulsion drops (15 wt% SFO) does not affect the formed structures in the case of P3-1-S. The comparison between Figures S2 and S7 leads to the analogous conclusions for P6-2-S.

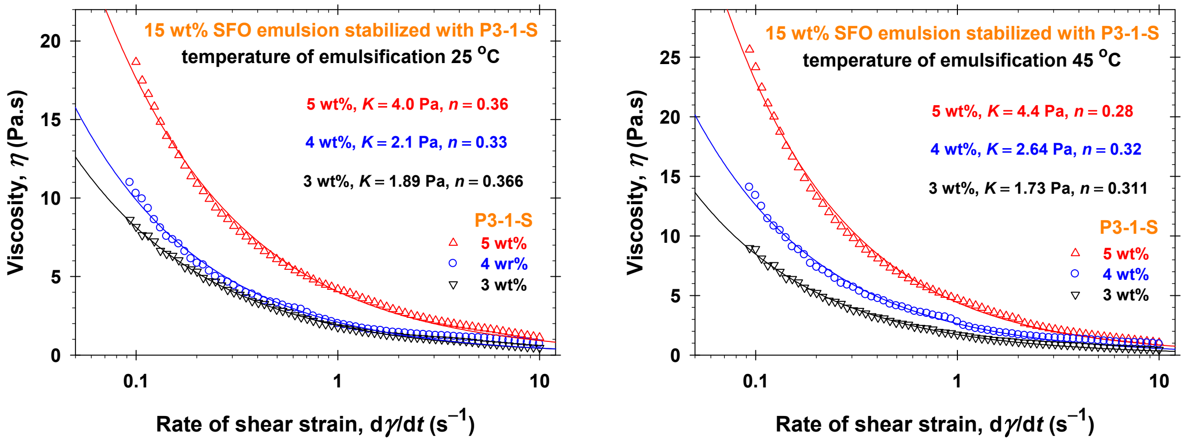

(a) **(b)**

Figure S6. Dependence of the viscosity, η, of 15 wt% SFO emulsions stabilized with P3-1-S on the shear rate, $d\gamma/dt$, and concentration of P3-1-S in aqueous phase measured at T = 25 °C: (**a**) 25 °C temperature of emulsification; (**b**) 45 °C temperature of emulsification. The solid lines correspond to the best-fit results

using the Ostwald–de Waele rheological model with the parameters listed in Table S2.

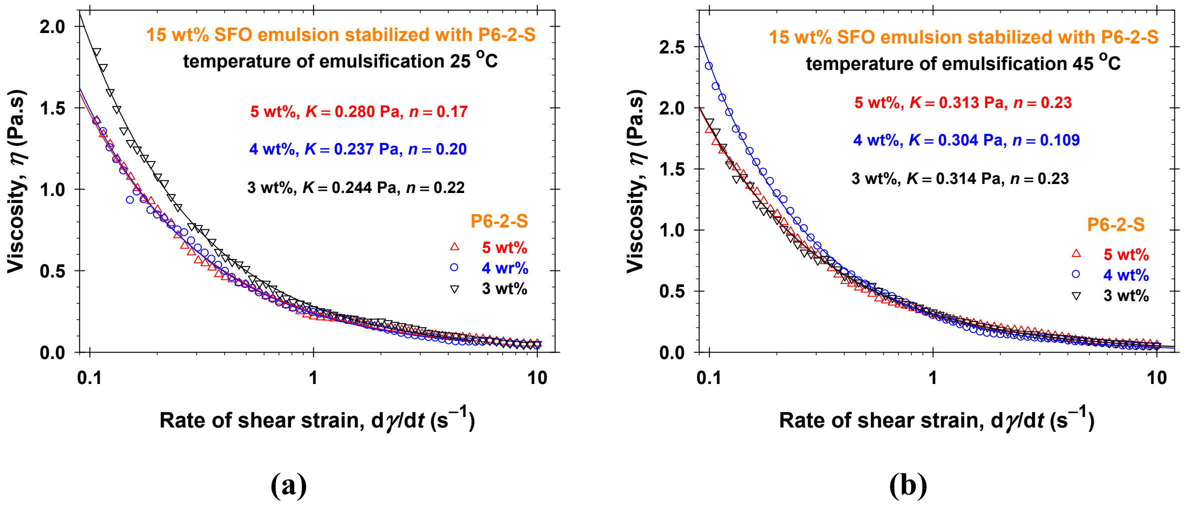

Figure S7. Dependence of the viscosity, η, of 15 wt% SFO emulsions stabilized with P6-2-S on the shear rate, $d\gamma/dt$, and concentration of P6-2-S in aqueous phase measured at T = 25 °C: (**a**) 25 °C temperature of emulsification; (**b**) 45 °C temperature of emulsification. The solid lines correspond to the best-fit results using the Ostwald–de Waele rheological model with the parameters listed in Table S2.

Table S3 shows the photographs of 15 wt% SFO emulsions stabilized with 3 wt%, 4 wt%, and 5 wt% PGE in the water phase taken three weeks after preparation in the case of T_{em} = 25 °C and T_{em} = 45 °C.

Table S3. Stability of emulsions containing 15 wt% SFO and different concentrations of PGE in the water phase, evaluated three weeks after preparation at both T_{em}.

T_{em} = 25 °C	T_{em} = 45 °C
P10-1-S	
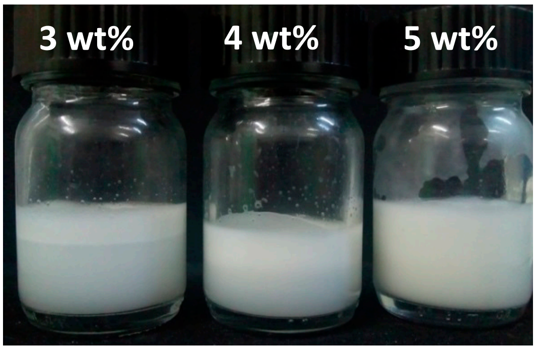	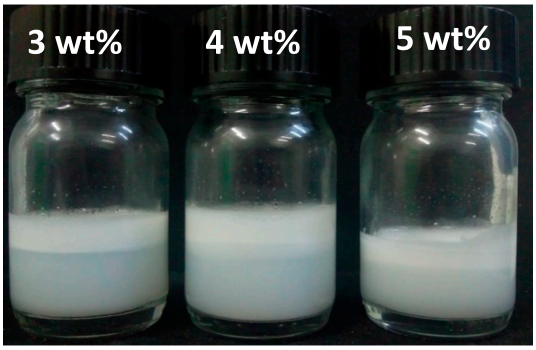
P10-2-P	

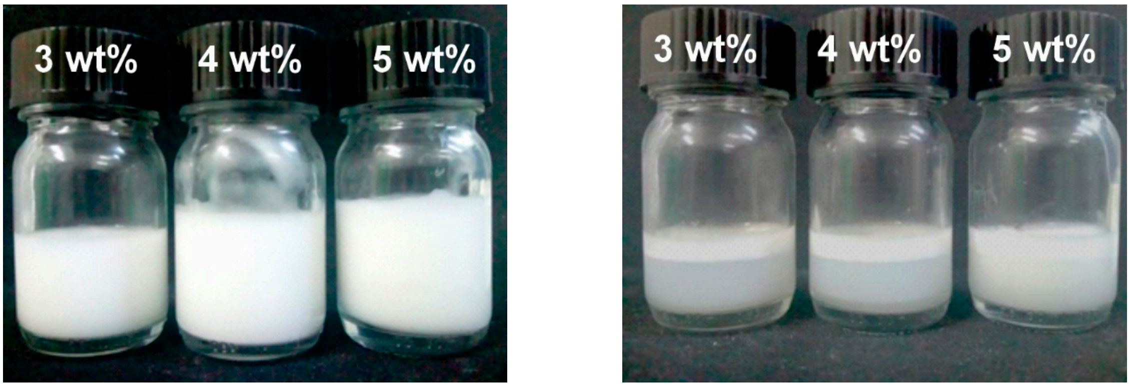

P3-1-S

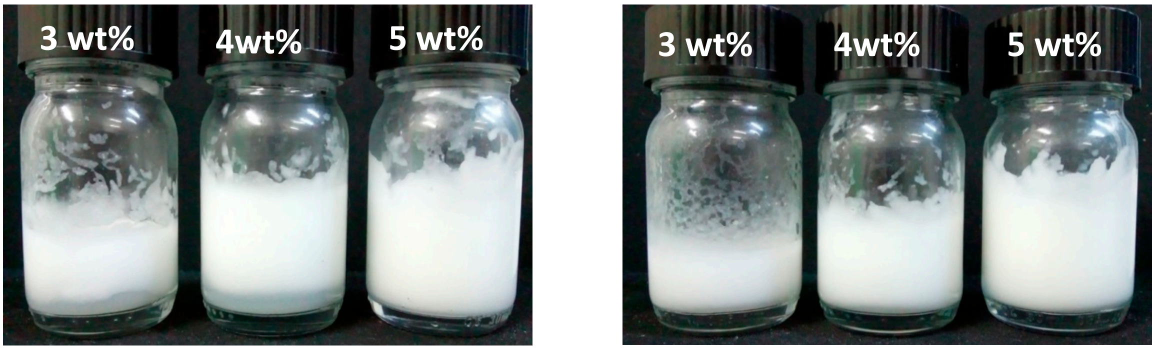

P6-2-S

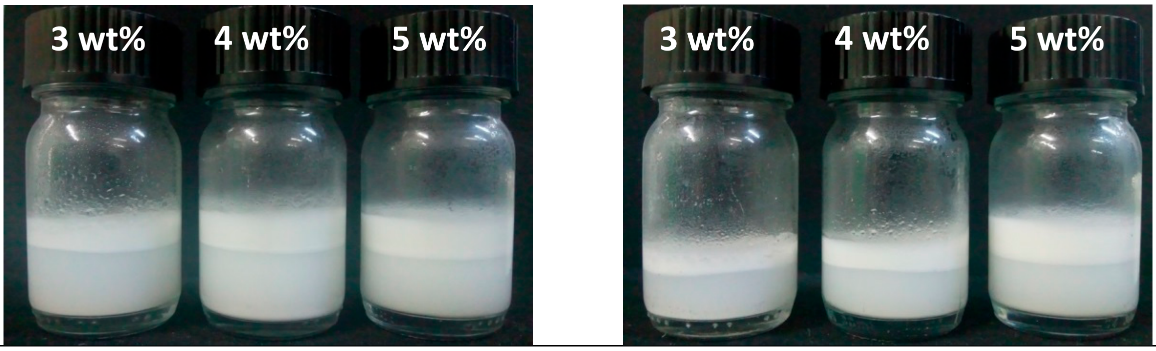

Table S3 shows that the stable emulsions are as follows: 5 wt% P10-1-S at T_{em} = 25 °C; 3, 4, and 5 wt% P10-2-P at T_{em} = 25 °C; 5 wt% P3-1-S at both temperatures of emulsification 25 °C and 45 °C. All studied emulsions stabilized with P6-2-S are unstable after three weeks of storage.

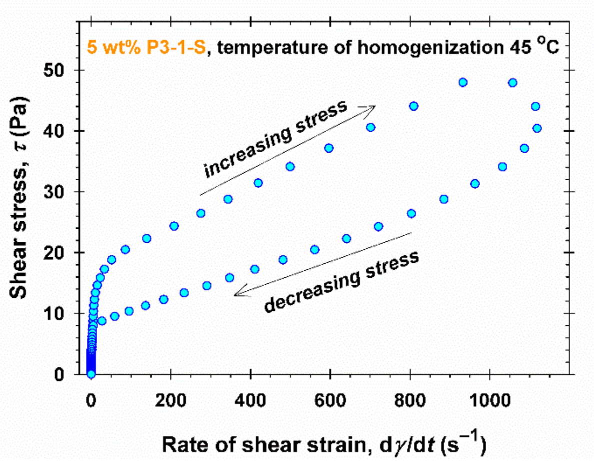

(a)

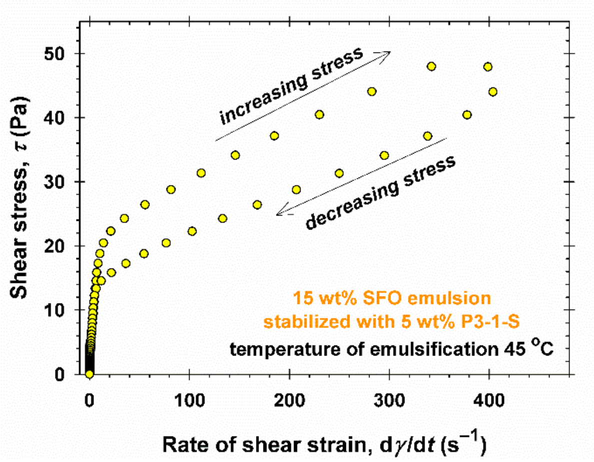

(b)

Figure S8. Thixotropic rheological experiments performed at 25 °C: (**a**) 5 wt% P3-1-S aqueous solutions, T_{hom} = 45 °C; (**b**) 15 wt% SFO emulsion stabilized with 5 wt% P3-1-S dissolved in the water phase, T_{em} = 45 °C.

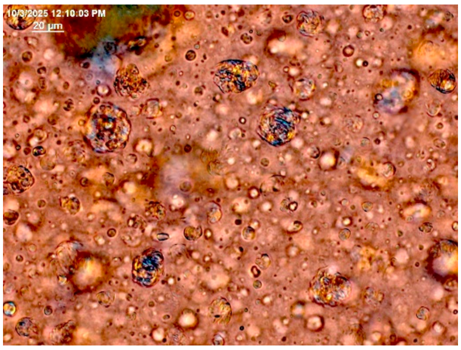

(a)

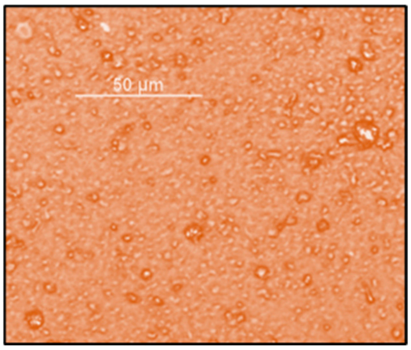

(b)

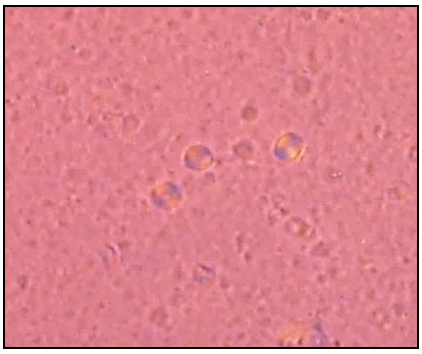

(c)

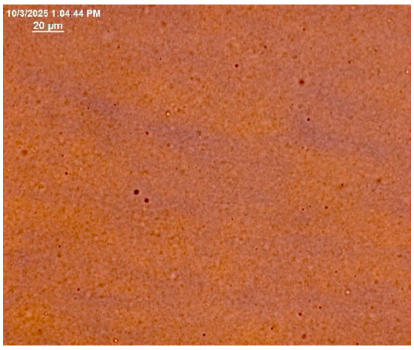

(d)

Figure S9. Microscopic images of 10 wt% PGE aqueous solutions in polarized light: (**a**) P3-1-S; (**b**) P6-2-S; (**c**) P10-1-S; and(**d**) P10-2-P.
